# Supplementary material for: Single Cell Transcriptomic and Chromatin Profiles Suggest Layer Vb Is the Only Layer With Shared Excitatory Cell Types in the Medial and Lateral Entorhinal Cortex
Source: Front Neural Circuits. 2022 Jan 26;15:806154. doi: 10.3389/fncir.2021.806154 (PMC8826650; doi:10.3389/fncir.2021.806154)
Supplement: Supplementary file 14 [file Data_Sheet_1.ZIP › LEC_Sup_vs_MEC_Sup_Motifs/homerResults.html]

LEC\_Sup\_vs\_MEC\_Sup\_Motifs// - Homer de novo Motif Results


# Homer *de novo* Motif Results (LEC\_Sup\_vs\_MEC\_Sup\_Motifs//)

Known Motif Enrichment Results  
Gene Ontology Enrichment Results  
If Homer is having trouble matching a motif to a known motif, try copy/pasting the matrix file into
STAMP  
More information on motif finding results: HOMER
| Description of Results
| Tips
  
Total target sequences = 13334  
Total background sequences = 19928  
\* - possible false positive  

|  |  |  |  |  |  |  |  |  |
| --- | --- | --- | --- | --- | --- | --- | --- | --- |
| Rank | Motif | P-value | log P-pvalue | % of Targets | % of Background | STD(Bg STD) | Best Match/Details | Motif File |
| 1 | G A T C G T C A A G T C C G T A A G T C A G T C A G T C G T C A A G T C C G A T | 1e-167 | -3.865e+02 | 42.59% | 31.19% | 221.8bp (249.5bp) | Egr2(Zf)/Thymocytes-Egr2-ChIP-Seq(GSE34254)/Homer(0.907) More Information \| Similar Motifs Found | motif file (matrix) |
| 2 | G A T C G A C T C T G A G C T A C G A T C G T A C T G A G A C T C T G A C A T G | 1e-120 | -2.777e+02 | 17.26% | 10.54% | 201.8bp (277.9bp) | MEF2B/MA0660.1/Jaspar(0.886) More Information \| Similar Motifs Found | motif file (matrix) |
| 3 | G T C A G C A T A C G T C G T A T C G A C G A T G T A C C G T A C A T G G T A C | 1e-74 | -1.715e+02 | 31.57% | 24.54% | 229.5bp (277.7bp) | Pdx1(Homeobox)/Islet-Pdx1-ChIP-Seq(SRA008281)/Homer(0.837) More Information \| Similar Motifs Found | motif file (matrix) |
| 4 | A C G T A C T G G A C T A G C T C G T A A C G T A G T C C G T A | 1e-62 | -1.428e+02 | 52.28% | 45.07% | 235.3bp (288.8bp) | GATA5/MA0766.2/Jaspar(0.904) More Information \| Similar Motifs Found | motif file (matrix) |
| 5 | A C T G A C T G A C T G A C T G A C T G A C T G A C T G A C T G A C T G A C T G | 1e-50 | -1.164e+02 | 30.81% | 25.04% | 252.8bp (260.4bp) | PB0097.1\_Zfp281\_1/Jaspar(0.899) More Information \| Similar Motifs Found | motif file (matrix) |
| 6 | G A T C A G T C G C T A A C G T C T A G T C A G G T A C C G T A C G T A A G T C | 1e-37 | -8.630e+01 | 8.70% | 5.90% | 203.8bp (247.5bp) | RFX3/MA0798.2/Jaspar(0.952) More Information \| Similar Motifs Found | motif file (matrix) |
| 7 | A T C G A T G C A G T C A C T G A G T C A T C G A G T C G T A C A C T G A G T C C T A G A G T C | 1e-33 | -7.811e+01 | 0.34% | 0.03% | 277.6bp (100.6bp) | PB0199.1\_Zfp161\_2/Jaspar(0.793) More Information \| Similar Motifs Found | motif file (matrix) |
| 8 | C G T A C A T G G T A C G C T A G C T A G C A T C G A T G C T A C G T A G A C T C G A T C T A G | 1e-31 | -7.331e+01 | 6.64% | 4.39% | 217.3bp (280.9bp) | Hmx1/MA0896.1/Jaspar(0.828) More Information \| Similar Motifs Found | motif file (matrix) |
| 9 | A G T C C G A T C T G A C G A T A C G T A C G T C T A G A G C T | 1e-30 | -6.984e+01 | 64.04% | 59.16% | 233.8bp (269.2bp) | FOXM1(Forkhead)/MCF7-FOXM1-ChIP-Seq(GSE72977)/Homer(0.802) More Information \| Similar Motifs Found | motif file (matrix) |
| 10 | G T A C C G T A A G T C G T C A A C G T A G T C C G T A C T A G | 1e-29 | -6.702e+01 | 21.91% | 18.05% | 235.8bp (274.9bp) | ZBTB18/MA0698.1/Jaspar(0.792) More Information \| Similar Motifs Found | motif file (matrix) |
| 11 | A C G T A C G T T G A C C T G A A G T C C T G A A G T C A C G T | 1e-25 | -5.887e+01 | 11.66% | 8.95% | 237.9bp (284.1bp) | Tbx21(T-box)/GM12878-TBX21-ChIP-Seq(Encode)/Homer(0.810) More Information \| Similar Motifs Found | motif file (matrix) |
| 12 | G A C T C T G A A G T C C G T A T C A G G C A T C G T A T C G A T C G A T A G C G T C A C T G A | 1e-22 | -5.097e+01 | 10.03% | 7.68% | 241.2bp (278.1bp) | FOXN3/MA1489.1/Jaspar(0.814) More Information \| Similar Motifs Found | motif file (matrix) |
| 13 | A G T C G T A C A C T G A G T C A C G T C G T A G T C A A C G T C G T A A G C T | 1e-20 | -4.773e+01 | 0.22% | 0.02% | 207.4bp (212.4bp) | Arid5a/MA0602.1/Jaspar(0.646) More Information \| Similar Motifs Found | motif file (matrix) |
| 14 | C G T A C G T A C G T A A G T C C G T A C G T A C T G A G A T C A G T C A C T G | 1e-20 | -4.674e+01 | 23.93% | 20.60% | 231.4bp (262.8bp) | PB0016.1\_Foxj1\_1/Jaspar(0.737) More Information \| Similar Motifs Found | motif file (matrix) |
| 15 | G C T A G C A T A G T C G C T A A G T C G C A T C A T G G C A T G C T A C G A T G A T C G C T A | 1e-19 | -4.420e+01 | 3.38% | 2.15% | 225.8bp (272.3bp) | DMRT3/MA0610.1/Jaspar(0.713) More Information \| Similar Motifs Found | motif file (matrix) |
| 16 | C G T A C T A G G A C T G C T A G A C T A G T C C A T G C T G A C T A G G T C A C G A T C G T A | 1e-17 | -4.055e+01 | 0.16% | 0.01% | 172.8bp (36.1bp) | GATA3(Zf)/iTreg-Gata3-ChIP-Seq(GSE20898)/Homer(0.623) More Information \| Similar Motifs Found | motif file (matrix) |
| 17 | A G T C A C G T A G T C A T G C C G T A A C T G C G T A A T C G A C T G A C G T | 1e-15 | -3.517e+01 | 0.34% | 0.08% | 205.2bp (183.4bp) | ZBTB26/MA1579.1/Jaspar(0.756) More Information \| Similar Motifs Found | motif file (matrix) |
| 18 | G T A C G T C A T G C A C T G A G A T C G C T A G C T A G C A T C T G A T A C G C G T A C A T G | 1e-15 | -3.494e+01 | 0.78% | 0.32% | 208.7bp (211.1bp) | SOX15/MA1152.1/Jaspar(0.755) More Information \| Similar Motifs Found | motif file (matrix) |
| 19 | G T A C G C T A C G A T G T A C G T C A C G T A G T A C G C T A T G A C G T A C G T C A C G T A | 1e-14 | -3.371e+01 | 18.70% | 16.15% | 235.6bp (271.6bp) | PB0122.1\_Foxk1\_2/Jaspar(0.612) More Information \| Similar Motifs Found | motif file (matrix) |
| 20 \* | C G T A C G T A C G A T A C G T C G T A A C T G C G T A C T A G A G T C C G T A | 1e-11 | -2.643e+01 | 2.69% | 1.84% | 198.3bp (293.6bp) | TLX2/MA1577.1/Jaspar(0.714) More Information \| Similar Motifs Found | motif file (matrix) |
| 21 \* | G T C A T G A C G T A C G C A T G T C A C G T A G A T C G A T C T G A C G A C T G C T A T A C G | 1e-9 | -2.121e+01 | 0.13% | 0.02% | 283.0bp (81.7bp) | CRX(Homeobox)/Retina-Crx-ChIP-Seq(GSE20012)/Homer(0.614) More Information \| Similar Motifs Found | motif file (matrix) |
| 22 \* | A C T G A G T C A C T G A C T G A G T C A C T G A C T G A G T C A C T G A C T G A G T C A C T G | 1e-9 | -2.081e+01 | 1.05% | 0.60% | 328.9bp (293.0bp) | PB0010.1\_Egr1\_1/Jaspar(0.722) More Information \| Similar Motifs Found | motif file (matrix) |
| 23 \* | C T A G A C G T A C G T C G T A C G T A C G T A A C G T A C G T | 1e-7 | -1.754e+01 | 36.76% | 34.50% | 245.0bp (296.7bp) | LMX1B/MA0703.2/Jaspar(0.714) More Information \| Similar Motifs Found | motif file (matrix) |
| 24 \* | C G T A C G T A C G A T A C G T A G T C C A T G C G T A C G T A | 1e0 | -1.476e-01 | 4.75% | 4.95% | 263.6bp (253.8bp) | LIN54/MA0619.1/Jaspar(0.783) More Information \| Similar Motifs Found | motif file (matrix) |
